# Cardiovascular Medication and Health Service Use in Individuals With Cancer: A Retrospective Population‐Based Cohort Study

**DOI:** 10.1002/cam4.70911

**Published:** 2025-05-09

**Authors:** Jin Quan Eugene Tan, Huah Shin Ng, Richard Woodman, Bogda Koczwara

**Affiliations:** ^1^ Flinders Health and Medical Research Institute College of Medicine and Public Health, Flinders University Adelaide South Australia Australia; ^2^ SA Pharmacy SA Health Adelaide South Australia Australia; ^3^ Department of Medical Oncology Flinders Medical Centre Adelaide South Australia Australia

**Keywords:** Australia, cancer, cardiovascular disease, health service utilisation

## Abstract

**Background:**

Cancer and cardiovascular disease (CVD) frequently coexist, but little is known about CVD medication use in cancer survivors. The aim of this study was to compare CVD medication and medical service use between individuals with and without cancer.

**Methods:**

Retrospective cohort study linking Australian National Health Survey 2020–2021 data from participants aged ≥ 25 years to medication dispensing, medical services, and death registry data via the Multi‐Agency Data Integration Project. Logistic regression was used to compare patterns of CVD medication use between cancer and non‐cancer groups and negative binomial regression to examine patterns of medical service utilisation by cancer and CVD status.

**Results:**

The analysis included 1828 individuals with a history of cancer (cancer survivors) and 7505 people without cancer. Although cancer survivors had a higher prevalence of CVD (31% vs. 13%) compared to people without cancer, there was no difference in the adjusted use of CVD medications (adjusted odds ratios: 1.15; 95% CI = 1.00–1.33) between these two groups. There was, however, an increased rate of health service use in those with cancer alone (adjusted rate ratios (aRR): 1.39; 95% CI = 1.29–1.50), those with CVD alone (aRR: 1.71; 95% CI = 1.63–1.80), and those with both conditions (aRR: 2.10; 95% CI = 1.97–2.25) compared to people without cancer or CVD.

**Conclusions:**

Despite having a higher prevalence of CVD and higher health service utilisation, the overall use of CVD medication did not differ between people with and without cancer. Cancer survivors with CVD had a higher rate of medical services use compared with persons with either condition alone or neither condition. Further research should explore the underlying reasons behind these data to inform strategies to mitigate the detrimental effects of comorbid CVD in cancer.

## Graphical abstract

This study found no difference in cardiovascular disease (CVD) medication use between cancer and the general population. Despite no differences in medication use, the study found higher health service utilisation in individuals with cancer and CVD, suggesting higher need burden and/or limited care integration.

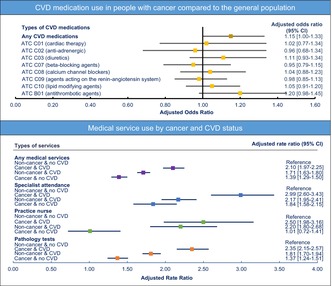

## 1 Introduction

Cardiovascular disease (CVD) is the leading cause of non‐cancer death among cancer survivors [1] with several population‐based studies of long‐term survivors demonstrating consistently higher CVD mortality across various cancer types compared to the general population [2, 3, 4]. The co‐existence of CVD and cancer can be explained by shared risk factors between cancer and CVD, including age, smoking status, a sedentary lifestyle, and poor dietary habits [5], and exposure to cardiotoxic cancer therapy may also increase the risk of CVD [6]. Although cardioprotective medications such as beta‐blockers, statins, and angiotensin‐converting enzyme inhibitors (ACEI) may also help attenuate cardiotoxicity associated with cancer treatment [7], their use in cancer survivors is inconsistent, with both underutilisation and higher utilisation being reported [8, 9, 10]. There is also little known about the utilisation of medical services in cancer survivors with CVD.

The aim of the study was to examine (a) patterns of CVD medication use between people with and without cancer, and (b) patterns of medical service use according to cancer and CVD status. We also assessed characteristics associated with CVD medication and medical service use in cancer and the general population.

## 2 Methods

### 2.1 Data Source

We used data from the most recent Australian National Health Survey (NHS) 2020–2021 conducted by the Australian Bureau of Statistics (ABS) linked to three administrative databases through the Multi‐Agency Data Integration Project [11]. The administrative databases included data from the Pharmaceutical Benefits Scheme (PBS), capturing medication dispensing records; data from the Medicare Benefits Schedule (MBS), capturing medical services usage; and death registrations, capturing death dates. The PBS and MBS are Australian Government funded schemes that provide subsidised prescription medications and medical services (e.g., out‐of‐hospital in doctor's consulting rooms) respectively, to eligible Australian citizens and permanent residents [12, 13]. Non‐subsidised medications (e.g., over‐the‐counter medications, vitamin supplements, complementary medicines, and medication written as ‘private’ prescriptions) and services paid entirely out‐of‐pocket (e.g., outside of Medicare or private health insurance) were not captured in our datasets. The NHS population consisted of randomly selected households across all Australian states and territories. Due to coronavirus disease 2019 pandemic, the NHS was deployed using an online self‐complete form for the 2020–2021 survey, in which 32,092 households were contacted, and 11,110 households responded (response rate = 35%) [14]. Excluded from the survey were non‐private dwellings (e.g., hotels, hospitals, nursing homes), very remote areas, discrete Aboriginal and Torres Strait Islander communities, and households where all usual residents were ≤ 18 years of age. The ABS employed a three‐stage deterministic linkage approach, linking 96.93% of the NHS 2020–2021 to the Person Linkage Spine (PLS) [15], which was then used to link the ABS data to the administrative databases. We accessed the de‐identified data at the individual level through the ABS secure DataLab [16].

### 2.2 Study Population

The study population consisted of all individuals aged ≥ 25 years in the survey with PLS and was followed for one‐year from the date of survey completion. People who died during the one‐year period were excluded from the analysis. To examine the prevalence of CVD medication use according to cancer status, we divided the study population into two groups: people with cancer (‘cancer survivors’ including people with active cancer and cancer in remission) and people without cancer (individuals with no cancer history) [17]. Information on cancer staging and diagnosis dates were not collected, and therefore, we included all people with cancer, with results reflecting the overall cancer survivorship journey. When examining the patterns of medical service use, we further divided each of the two study populations by CVD status.

### 2.3 Outcome Measures

#### 2.3.1 1‐Year CVD Medications Use

Dispensing of CVD medications was identified in the PBS data by using the Anatomical Therapeutical Chemical Classification (ATC) at the 2nd level to consider cardiac therapy, anti‐adrenergic, diuretics, beta‐blockers, calcium channel blockers, agents acting on the renin‐angiotensin system, lipid‐modifying agents, and anti‐thrombotic agents (Table S1). CVD medication use was defined as the dispensing of any CVD medications within 1 year from the survey completion date, and then by the listed subtypes of ATC categories.

#### 2.3.2 1‐Year Medical Services Use

MBS captures a range of government‐subsidised medical services provided out‐of‐hospital. Medical services were classified using the Medicare Broad Types of Service (BTOS) classification system, which comprised 18 distinct categories [18]. The incidence of medical service use was defined as the record of any BTOS within 1 year from the survey completion date. We included all medical services except for two categories related to dental services as they were classified as non‐Medicare and item codes related to bulk billing incentives. We also compared the rates of medical service use by each BTOS category except for three categories due to low frequency counts (obstetrics, radiotherapy/therapeutic nuclear medicine, and assistance at operations) (Table S2).

### 2.4 Covariates: Sociodemographic, Lifestyle Factors, and Comorbidities

Sociodemographic variables included age, sex, marital status, birth country, geographic location (defined by the Australian Standard Geographical Classification Remoteness Index and categorised according to remoteness as major cities, inner regionals, outer regionals, remote and very remote areas) [19], educational attainment, employment status, and socioeconomic status (measured by weekly personal income). Health and lifestyle‐related variables comprised body mass index, smoking habits, alcohol consumption, dietary habits, physical activity levels, self‐reported CVD, and the total number of other self‐reported current health conditions (comorbidities).

### 2.5 Statistical Analysis

Descriptive statistics including frequency (percentages) for categorical variables were used to describe the study population characteristics, with differences in frequency by cancer status assessed using chi‐squared tests. We compared the odds of CVD medication use (defined as a binary outcome: yes/no) with cancer status as the exposure variable and adjusted for sociodemographic, lifestyle factors, and the number of comorbidities in the logistic regression analysis. Two levels of adjustment were performed (Model A and Model B): with and without adjustment for self‐reported CVD status. Results were presented as adjusted odds ratios (aORs) and 95% confidence intervals (CIs).

Patterns of medical service utilisation according to both cancer and CVD status (determined by self‐reported current CVD and/or a dispensing record of CVD medication) were assessed by considering the number of medical services utilised to be distributed as count variables (i.e., number of all medical services) and therefore analysed using negative binomial regression. Findings are reported as adjusted rate ratios (aRRs) and 95% CIs with the same adjustment variables as used for the analysis of CVD medication utilisation, except for CVD status since this was used as a stratification variable. The crude rates of each category of medical services utilisation were reported as the number of occurrences per 100 person‐years of follow‐up by cancer and CVD status.

As a sensitivity analysis, we also assessed the CVD medication and medical service utilisation by excluding people aged less than 50 years in the logistic regression and negative binomial regression analysis.

Predictive factors for CVD medication and medical service utilisation were assessed separately for the cancer and non‐cancer groups. A separate multivariable prediction model was used for each analysis (cancer and non‐cancer groups) with pre‐specified variables including sociodemographic, lifestyle factors, and comorbidities, and for the cancer group only, an additional variable on their disease status (current cancer or non‐current). All analyses were conducted using R version‐4.2.1. Statistical significance was defined using a 2‐sided type 1 error rate of alpha = 0.05.

## 3 Results

### 3.1 Study Population Characteristics

**FIGURE 1:**
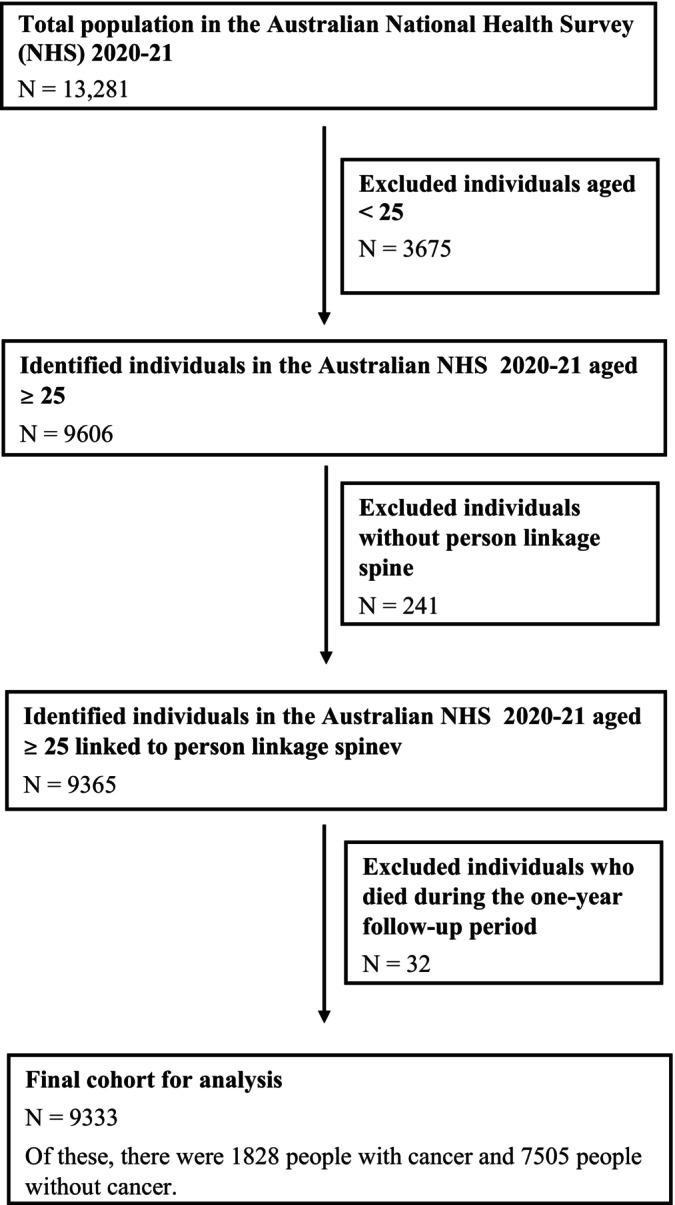
Flow diagram of the cohort selection from the Australian National Health Survey 2020–2021.

Of 9606 individuals aged ≥ 25 years who participated in the NHS 2020–2021, we excluded 241 (2.5%) respondents from the analysis due to failure of linkage to the PLS, and an additional 32 (0.3%) who died during the study period (Figure 1). The final analysis included a total of 9333 respondents: 1828 people with cancer and 7505 without cancer (Table 1). A greater proportion of people with cancer were older (57% vs. 21% aged ≥ 65 years), born in Australia (77% vs. 65%), unemployed (60% vs. 33%), residing in inner/outer regionals (i.e., areas that fall outside of major cities: 40% vs. 34%), and had a lower education level (34% vs. 27% without non‐school qualifications) and a lower socioeconomic status (19% vs. 27% in the highest equivalised personal weekly income level) than people without cancer. Cancer survivors were also more likely to have a history of smoking (48% vs. 40%), meet the recommended guidelines for fruit and/or vegetables consumption (56% vs. 48%), and physical activity (35% vs. 29%), and have a higher burden of comorbidities (85% vs. 72% with ≥ 1 concurrent conditions), and a higher prevalence of self‐reported CVD (31% vs. 13%) compared to people without cancer.

**TABLE 1:** Characteristics of the study population by cancer status.

Characteristics	Level	Cancer	Non‐cancer	*p*
		*N* = 1828 (%)	*N* = 7505 (%)	
Sex	Female	1009 (55.2)	4057 (54.1)	0.395
	Male	819 (44.8)	3448 (45.9)	
Age group	25–34	19 (1.0)	1340 (17.9)	< 0.001
	35–49	200 (10.9)	2516 (33.5)	
	50–64	567 (31.0)	2097 (27.9)	
	65 and over	1042 (57.0)	1552 (20.7)	
Country of birth	Australia	1403 (76.8)	4908 (65.4)	< 0.001
	Others	425 (23.2)	2597 (34.6)	
Geographical location	Inner/outer regionals	728 (39.8)	2569 (34.2)	< 0.001
	Major cities	1085 (59.4)	4852 (64.7)	
	Remote	15 (0.8)	84 (1.1)	
Marital status	Married	1097 (60.0)	4288 (57.1)	< 0.001
	Never married	192 (10.5)	1779 (23.7)	
	Separated/widowed/divorced	539 (29.5)	1438 (19.2)	
Education level	Bachelor	373 (20.4)	1987 (26.5)	< 0.001
	Certificate	348 (19.0)	1345 (17.9)	
	Diploma	206 (11.3)	874 (11.6)	
	No non‐school qualification	613 (33.5)	2015 (26.8)	
	Postgraduate	234 (12.8)	1124 (15.0)	
	Not known	54 (3.0)	160 (2.1)	
Employment status	Employed	725 (39.7)	5063 (67.5)	< 0.001
	Unemployed/not in labour force	1103 (60.3)	2442 (32.5)	
Socioeconomic status (equivalised personal weekly income^a^)	Decile 1–2 (lowest)	199 (10.9)	980 (13.1)	< 0.001
	Decile 3–4	476 (26.0)	1205 (16.1)	
	Decile 5–6	436 (23.9)	1487 (19.8)	
	Decile 7–8	356 (19.5)	1760 (23.5)	
	Decile 9–10 (highest)	344 (18.8)	2025 (27.0)	
	Not known	17 (0.9)	48 (0.6)	
Body mass index	Normal	618 (33.8)	2728 (36.3)	0.005
	Underweight	21 (1.1)	103 (1.4)	
	Overweight	669 (36.6)	2512 (33.5)	
	Obese	478 (26.1)	1896 (25.3)	
	Missing	42 (2.3)	266 (3.5)	
Smoking status	Current smoker	120 (6.6)	647 (8.6)	< 0.001
	Ex‐smoker	751 (41.1)	2355 (31.4)	
	Never smoked	957 (52.4)	4503 (60.0)	
Met recommended vegetable/fruits guidelines^b^	Met both	150 (8.2)	502 (6.7)	< 0.001
	Met either	864 (47.3)	3062 (40.8)	
	Not met/missing	814 (44.5)	3941 (52.5)	
Alcohol intake^c^	Not applicable	344 (18.8)	1559 (20.8)	< 0.001
	Everyday	166 (9.1)	324 (4.3)	
	1 to 3 days a month	266 (14.6)	1396 (18.6)	
	2 to 6 days a week	802 (43.9)	3037 (40.5)	
	Less than once a month	221 (12.1)	1078 (14.4)	
	Not known	29 (1.6)	111 (1.5)	
Met physical activity guidelines^d^	No	1192 (65.2)	5369 (71.5)	< 0.001
	Yes	636 (34.8)	2136 (28.5)	
Number of health conditions (excl cancer & CVD)	0	276 (15.1)	2076 (27.7)	< 0.001
	1–2	955 (52.2)	3927 (52.3)	
	3–4	501 (27.4)	1303 (17.4)	
	≥ 5	96 (5.3)	199 (2.7)	
Presence of CVD (self‐reported)	No	1264 (69.1)	6534 (87.1)	< 0.001
	Yes	564 (30.9)	971 (12.9)	

Abbreviation: CVD, cardiovascular disease.

^a^ Higher deciles signifying greater weekly personal income.

^b^ Compliance with recommended daily fruit and vegetable intake based on the Australian Dietary Guidelines 2013.

^c^ Frequency of alcohol use in the past 12 months.

^d^ Evaluated using the Physical Activity Guidelines 2014.

### 3.2 Association Between Cancer Status and CVD Medication Use

**FIGURE 2:**
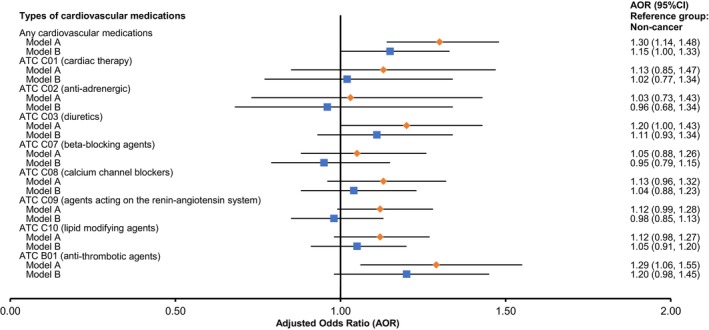
The odds of dispensing cardiovascular medications between people with and without cancer (reference category). AOR, adjusted odds ratio; ATC, Anatomical Therapeutic Chemical; CI, confidence interval. Model A: The logistic regression model was adjusted for sociodemographic characteristics (sex, age group, marital status, country of birth, geographical location, education level, employment status, socioeconomic status), and lifestyle factors (body mass index, smoking status, alcohol intake, vegetable/fruit intake, and physical activity), as well as the number of other current health conditions. Model B: The model was adjusted using the same adjustment variables as model A, with the addition of self‐reported cardiovascular disease status.

In the analysis using partial adjustment, cancer survivors had an overall higher odds (Model A—aOR: 1.30; 95% CI = 1.14–1.48) of having a dispensing record for a CVD medicine than persons without cancer, but the difference no longer remained significant after adjusting for self‐reported CVD status (Model B—aOR: 1.15; 95% CI = 1.00–1.33) (Figure 2). For individual types of ATC categories, there were also no significant differences between the two groups in the relative dispensing of cardiac therapy, antiadrenergic, diuretics, beta‐blockers, calcium channel blockers, agents acting on the renin‐angiotensin system, and the lipid‐modifying agents. An exception to this trend was for anti‐thrombotic usage, for which the aOR was significantly higher in people with cancer compared to those without cancer (Model A—aOR: 1.29; 95% CI = 1.06–1.55), but this association also no longer remained after adjusting for self‐reported CVD status (Model B—aOR: 1.20; 95% CI = 0.98–1.45). The proportion of CVD medication use by cancer status is shown in Table S3.

The direction of findings from the sensitivity analysis (by excluding those aged < 50 years old) was consistent with that of the main findings (Table S4).

### 3.3 Medical Services

**FIGURE 3:**
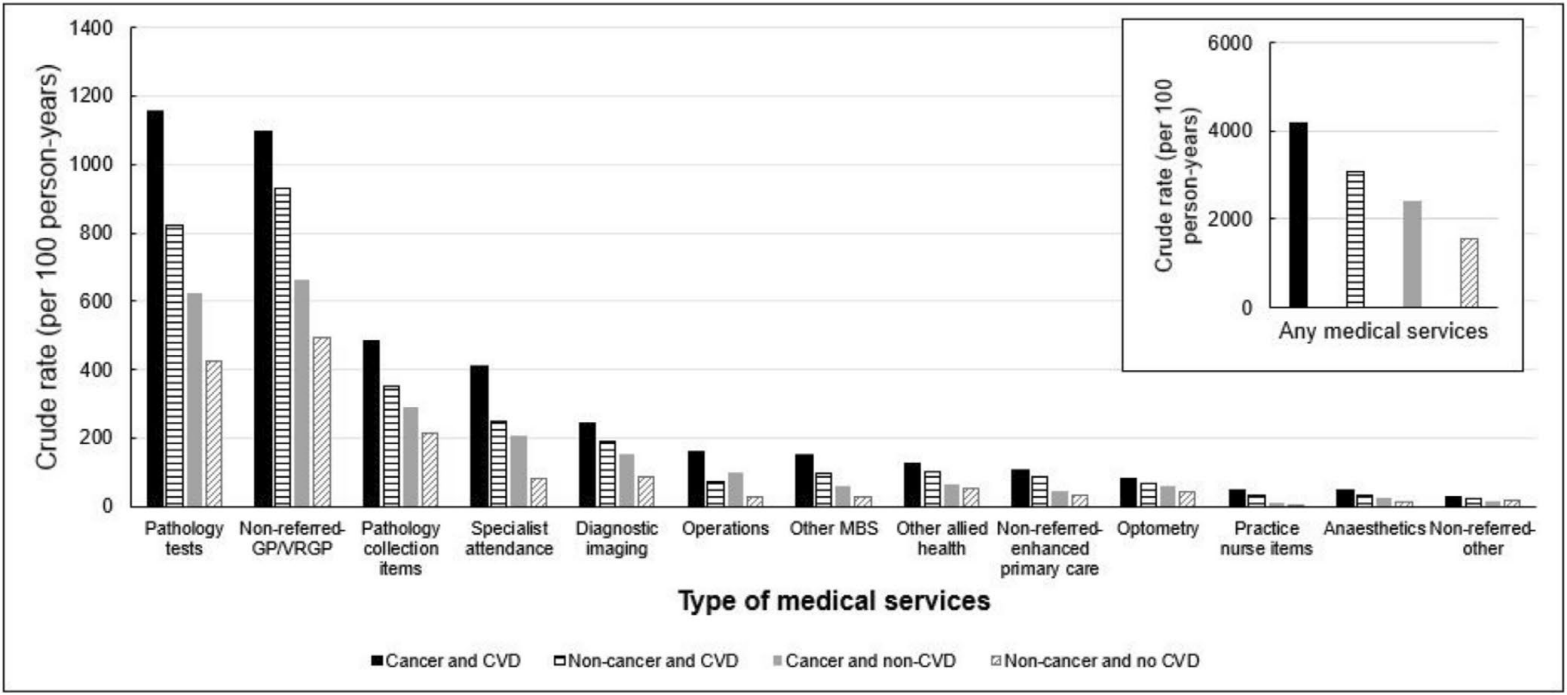
Rates of medical services by cancer and cardiovascular disease (CVD) status. CVD, cardiovascular disease; GP, general practitioner; VRGP, vocationally registered general practitioner. Crude rate was calculated using the formula: (total number of medical services for each broad types of services by cancer and CVD status/total person years of follow‐up by cancer and CVD status) × 100. The results for the ‘any medical services’ were presented in the right square box with a separate y‐axis value (i.e., with a 10 times higher y‐axis value than the remaining types of medical services). CVD status was determined by self‐reported as having a current CVD and/or with a dispensing record of CVD medications within 1 year from the date of survey completion. The number of people in each group is as follows: Cancer and CVD (*n* = 1141/9333; 12%), non‐cancer and CVD (*n* = 2477/9333; 27%), cancer and no CVD (*n* = 687/9333; 7%) and non‐cancer and no CVD (*n* = 5028/9333; 54%).

The crude rate of medical service use was highest in people with cancer and CVD (4197/100 person‐years), when compared to those with CVD alone (3079/100 person‐years), those with cancer alone (2399/100 person‐years), and those with neither condition (1543/100 person‐years) (Figure 3; Table S5). When analyzing by each health service, those with cancer and CVD consistently demonstrated the highest crude rates across all types of services.

When compared to people without cancer and CVD, there was a relative increase in the use of medical services in those with cancer alone (aRR: 1.39; 95% CI = 1.29–1.50), those with CVD alone (aRR: 1.71; 95% CI = 1.63–1.80), and those with both conditions (aRR: 2.10; 95% CI = 1.97–2.25) (Table 2). Similar patterns were seen across virtually all individual types of medical services whereby those with both conditions had the highest rates of health service utilisation, followed by those with CVD alone and those with cancer alone, when compared to people with neither condition.

**TABLE 2:** Medical service use by cancer and cardiovascular disease status.

Medical service categories by cancer and comorbidity status	Adjusted rate ratio^a^ (95% confidence interval)
Any medical services	
Non‐cancer, no CVD	Reference
Cancer & CVD	2.10 (1.97–2.25)*
Non cancer & CVD	1.71 (1.63–1.80)*
Cancer & no CVD	1.39 (1.29–1.50)*
By types of services	
Other medicare benefits schedule items	
Non‐cancer, no CVD	Reference
Cancer & CVD	3.68 (3.10–4.37)*
Non‐cancer & CVD	2.86 (2.50–3.28)*
Cancer & no CVD	1.72 (1.40–2.11)*
Specialist attendance	
Non‐cancer, no CVD	Reference
Cancer & CVD	2.99 (2.60–3.43)*
Non‐cancer & CVD	2.17 (1.95–2.41)*
Cancer & no CVD	1.84 (1.58–2.15)*
Operations	
Non‐cancer, no CVD	Reference
Cancer & CVD	2.64 (2.26–3.08)*
Non‐cancer & CVD	1.51 (1.33–1.71)*
Cancer & no CVD	2.18 (1.84–2.59)*
Practice nurse items	
Non‐cancer, no CVD	Reference
Cancer & CVD	2.50 (1.98–3.16)*
Non‐cancer & CVD	2.20 (1.80–2.68)*
Cancer & no CVD	1.01 (0.72–1.41)
Pathology tests	
Non‐cancer, no CVD	Reference
Cancer & CVD	2.35 (2.15–2.57)*
Non‐cancer & CVD	1.81 (1.70–1.94)*
Cancer & no CVD	1.37 (1.24–1.51)*
Pathology collection items	
Non‐cancer, no CVD	Reference
Cancer & CVD	2.12 (1.96–2.30)*
Non‐cancer & CVD	1.64 (1.54–1.74)*
Cancer & no CVD	1.33 (1.21–1.45)*
Diagnostic imaging	
Non‐cancer, no CVD	Reference
Cancer & CVD	2.04 (1.82–2.29)*
Non‐cancer & CVD	1.80 (1.64–1.96)*
Cancer & no CVD	1.48 (1.30–1.68)*
Anaesthetics	
Non‐cancer, no CVD	Reference
Cancer & CVD	1.99 (1.59–2.49)*
Non‐cancer & CVD	1.96 (1.64–2.35)*
Cancer & no CVD	1.41 (1.08–1.83)*
Other allied health	
Non‐cancer, no CVD	Reference
Cancer & CVD	1.91 (1.48–2.46)*
Non‐cancer & CVD	1.77 (1.47–2.13)*
Cancer & no CVD	1.22 (0.92–1.63)
Non‐referred—enhanced primary care	
Non‐cancer, no CVD	Reference
Cancer & CVD	1.86 (1.61–2.15)*
Non‐cancer & CVD	1.78 (1.59–1.99)*
Cancer & no CVD	1.12 (0.94–1.34)
Non‐referred—GP/VRGP	
Non‐cancer, no CVD	Reference
Cancer & CVD	1.65 (1.56–1.75)*
Non‐cancer & CVD	1.53 (1.46–1.60)*
Cancer & no CVD	1.20 (1.13–1.28)*
Non‐referred—other	
Non‐cancer, no CVD	Reference
Cancer & CVD	1.57 (1.10–2.24)*
Non‐cancer & CVD	1.51 (1.17–1.96)*
Cancer & no CVD	0.99 (0.67–1.49)
Optometry	
Non‐cancer, no CVD	Reference
Cancer & CVD	1.25 (1.14–1.38)*
Non‐cancer & CVD	1.20 (1.11–1.29)*
Cancer & no CVD	1.10 (0.99–1.23)

*Note:* CVD status was determined by self‐reported as having a current CVD and/or with a dispensing record of CVD medications within 1 year from the date of survey completion. Number of people in each group as follows: Non‐cancer and no CVD (*n* = 5028/9333; 54%), cancer and CVD (*n* = 1141/9333; 12%), non‐cancer and CVD (*n* = 2477/9333; 27%) and cancer and no CVD (*n* = 687/9333; 7%).

Abbreviations: CVD, cardiovascular disease; GP, general practitioner; VRGP, vocationally registered general practitioner.

^a^ The negative binomial regression was adjusted for sociodemographic characteristics (sex, age group, marital status, country of birth, geographical location, education level, employment status, socioeconomic status), and lifestyle factors (body mass index, smoking status, alcohol intake, vegetable/fruit intake, and physical activity), as well as the number of other current health conditions (excluding cancer).

* *p*‐value < 0.05.

The direction of findings from the sensitivity analysis (by excluding those aged < 50 years old) was consistent with that of the main findings (Table S6).

### 3.4 Predictors of CVD Medication and Medical Service Utilisation

People who were male, older (≥ 65 vs. 25–34 years), unemployed, and obese/overweight and having a lower education attainment (no non‐school qualification vs. postgraduate), ≥ 1 comorbidities, and a self‐reported CVD were more likely to have been dispensed any CVD medicine than their respective counterparts in both cancer and non‐cancer groups (Table 3). People who were having CVD and other comorbidities also had higher rates of medical service utilisation (Table 3). People who were residing outside of major cities and those with regular consumption of alcohol and actively smoking were less likely to access medical services compared to their respective counterparts in both cancer and non‐cancer groups. In the cancer group, while those with a current cancer status (versus non‐current) were more likely to use medical services, there was no difference in CVD medication use by cancer status.

**TABLE 3:** Multivariable prediction models examining independent associations between individual characteristics and the dispensing of cardiovascular medications and medical service use in people with and without cancer.

Characteristics	Cardiovascular medication use		Medical service use	
	Adjusted odds ratio (95% CI)	Adjusted odds ratio (95% CI)	Adjusted rate ratio (95% CI)	Adjusted rate ratio (95% CI)
	Cancer	Non‐cancer	Cancer	Non‐cancer
Sex				
Female	Reference	Reference	Reference	Reference
Male	1.37 (1.04–1.80)*	1.29 (1.12–1.49)*	0.93 (0.86–1.01)	0.71 (0.68–0.74)*
Age group				
25–34	Reference	Reference	Reference	Reference
35–49	0.77 (0.23–3.17)	2.44 (1.85–3.28)*	0.95 (0.65–1.36)	0.94 (0.88–1.00)*
50–64	2.04 (0.64–8.13)	7.12 (5.39–9.53)*	1.07 (0.73–1.51)	1.01 (0.94–1.09)
65 and over	6.72 (2.05–27.29)*	22.74 (16.68–31.39)*	1.38 (0.94–1.97)	1.23 (1.13–1.35)*
Country of birth				
Australia	Reference	Reference	Reference	Reference
Others	1.22 (0.91–1.65)	0.94 (0.82–1.09)	0.97 (0.90–1.06)	1.01 (0.97–1.06)
Geographical location				
Major cities	Reference	Reference	Reference	Reference
Inner/outer regionals	0.95 (0.73–1.23)	0.91 (0.79–1.04)	0.79 (0.73–0.85)*	0.81 (0.77–0.84)*
Remote	1.05 (0.29–3.84)	1.26 (0.71–2.17)	0.52 (0.35–0.79)*	0.71 (0.59–0.87)*
Marital status				
Married	Reference	Reference	Reference	Reference
Never married	0.96 (0.63–1.47)	0.73 (0.60–0.88)	1.01 (0.89–1.14)	0.90 (0.85–0.95)*
Separated/widowed/divorced	0.80 (0.60–1.06)	0.94 (0.80–1.10)	1.00 (0.92–1.08)	0.96 (0.91–1.02)
Education level				
Postgraduate	Reference	Reference	Reference	Reference
Bachelor	1.08 (0.69–1.69)	1.26 (1.00–1.59)	1.00 (0.89–1.14)	0.96 (0.90–1.03)
Diploma	1.45 (0.87–2.41)	1.23 (0.94–1.60)	1.09 (0.94–1.27)	1.00 (0.92–1.08)
Certificate	1.49 (0.93–2.40)	1.39 (1.09–1.78)*	1.09 (0.95–1.25)	1.00 (0.92–1.08)
No non‐school qualification	1.84 (1.19–2.85)*	1.63 (1.30–2.06)*	1.06 (0.93–1.19)	0.91 (0.85–0.98)*
Not known	2.82 (1.24–6.77)*	1.32 (0.85–2.06)	1.28 (1.02–1.61)*	0.93 (0.81–1.09)
Employment status				
Employed	Reference	Reference	Reference	Reference
Unemployed/not in labour force	1.58 (1.14–2.18)*	1.33 (1.12–1.59)*	1.10 (0.99–1.21)	1.05 (0.99–1.11)
Socioeconomic status (equivalised personal weekly income^a^)				
Decile 9–10 (highest)	Reference	Reference	Reference	Reference
Decile 7–8	0.95 (0.63–1.42)	1.16 (0.96–1.41)	1.07 (0.95–1.21)	1.05 (0.99–1.12)
Decile 5–6	1.09 (0.72–1.66)	1.07 (0.87–1.33)	1.07 (0.94–1.21)	1.06 (0.99–1.13)
Decile 3–4	1.51 (0.96–2.37)	1.20 (0.94–1.52)	0.97 (0.85–1.11)	1.10 (1.02–1.19)*
Decile 1–2 (lowest)	0.87 (0.52–1.44)	1.06 (0.82–1.35)	1.05 (0.91–1.22)	1.01 (0.93–1.09)
Not known	0.56 (0.17–1.96)	0.73 (0.33–1.53)	0.79 (0.55–1.17)	1.26 (0.98–1.65)
Body mass index				
Normal	Reference	Reference	Reference	Reference
Underweight	0.70 (0.24–1.99)	0.56 (0.28–1.06)	0.74 (0.53–1.05)	0.95 (0.80–1.14)
Overweight	2.13 (1.59–2.87)*	1.43 (1.22–1.67)*	0.94 (0.86–1.02)	0.95 (0.91–1.00)
Obese	3.29 (2.35–4.63)*	2.48 (2.09–2.95)*	0.93 (0.85–1.03)	0.99 (0.93–1.04)
Missing	2.79 (1.23–6.40)*	1.85 (1.27–2.67)*	0.80 (0.63–1.03)	1.21 (1.08–1.36)*
Smoking status				
Never smoked	Reference	Reference	Reference	Reference
Ex smoker	1.03 (0.79–1.34)	0.95 (0.82–1.10)	0.90 (0.84–0.97)*	1.08 (1.03–1.13)*
Current smoker	1.27 (0.76–2.13)	1.19 (0.94–1.50)	0.82 (0.70–0.95)*	0.88 (0.82–0.96)*
Met recommended vegetable/fruits guidelines^b^				
Met both	Reference	Reference	Reference	Reference
Met either	0.96 (0.60–1.52)	0.86 (0.66–1.13)	0.98 (0.86–1.12)	1.05 (0.96–1.14)
Not met/missing	1.11 (0.69–1.77)	0.92 (0.71–1.20)	0.97 (0.85–1.11)	0.99 (0.91–1.08)
Alcohol intake^c^				
Not applicable	Reference	Reference	Reference	Reference
Everyday	1.15 (0.68–1.95)	0.97 (0.71–1.34)	0.89 (0.77–1.03)	0.88 (0.79–0.99)*
2–6 days a week	0.99 (0.70–1.40)	0.80 (0.67–0.95)*	0.84 (0.76–0.93)*	0.93 (0.87–0.98)*
1–3 days a month	0.69 (0.45–1.07)	0.73 (0.59–0.90)*	0.94 (0.83–1.07)	0.94 (0.88–1.01)
Less than once a month	1.55 (0.98–2.47)	0.86 (0.69–1.07)	0.92 (0.81–1.05)	1.02 (0.95–1.09)
Not known	0.81 (0.29–2.21)	1.21 (0.73–1.98)	0.95 (0.71–1.28)	0.94 (0.79–1.13)
Met physical activity guidelines^d^				
Yes	Reference	Reference	Reference	Reference
No	1.24 (0.95–1.62)	1.12 (0.96–1.30)	1.11 (1.02–1.20)*	0.99 (0.95–1.04)
Number of health conditions (excl cancer & CVD)				
0	Reference	Reference	Reference	Reference
1–2	1.93 (1.37–2.73)*	1.37 (1.17–1.61)*	1.00 (0.90–1.11)	1.33 (1.27–1.40)*
3–4	2.13 (1.43–3.18)*	1.82 (1.49–2.24)*	1.23 (1.10–1.39)*	1.66 (1.56–1.77)*
≥ 5	1.59 (0.81–3.19)	2.34 (1.54–3.54)*	1.50 (1.25–1.80)*	2.01 (1.76–2.30)*
Presence of CVD^e^				
No	Reference	Reference	Reference	Reference
Yes	15.34 (10.57–22.84)*	14.71 (11.78–18.51)*	1.48 (1.36–1.61)*	1.73 (1.65–1.83)*
Cancer status				
Non‐current	Reference	N/A	Reference	N/A
Current cancer	1.28 (0.86–1.90)		1.73 (1.55–1.93)*	

Abbreviations: CI, confidence interval; CVD, cardiovascular disease; N/A, not applicable.

^a^ Higher deciles signifying greater weekly personal income.

^b^ Compliance with recommended daily fruit and vegetable intake based on the Australian Dietary Guidelines 2013.

^c^ Frequency of alcohol use in the past 12 months.

^d^ Evaluated using the Physical Activity Guidelines 2014.

^e^ Self‐reported CVD status for the CVD medication dispensing outcome; Self‐reported CVD status and/or the dispensing of any CVD medications for medical services utilization outcome.

* *p*‐value < 0.05.

## 4 Discussion

In this study, while there was a higher prevalence of CVD in people with cancer than people without cancer, likely reflecting a greater proportion of people with cancer who were older, no difference was found in the odds of dispensing for CVD medications between the two groups after accounting for self‐reported CVD, sociodemographic, and lifestyle factors. There was, however, an increased relative usage of medical services in people with cancer and comorbid CVD compared to persons without CVD.

Our findings on CVD medication use are consistent with observations from others. A single‐centre Australian study of 69 people with cancer and 251 without cancer found no significant differences in the usage of beta‐blocker and ACEI/angiotensin receptor antagonist (ARB) between the two groups, although cancer patients had a lower odds of using statins and anti‐platelets [8]. A Swiss study of cancer (*n* = 1981) and non‐cancer patients (*n* = 1981) with acute myocardial infarction demonstrated that although there were no significant differences in the use of ACEI/ARB, beta‐blockers, nitrate, other antiplatelet and antithrombotic (aspirin and heparins) between the two groups, cancer patients were less likely to receive a P2Y12 inhibitor (an antiplatelet) and statins [20]. Also, a US study with 5,012,721 respondents reported that patients with CVD and cancer had lower utilisation of anti‐hypertensives, lipid‐modifying therapies, and aspirin compared to the general population with CVD [9]. Our study adds a unique contribution to this evidence as previous studies focused on either self‐reported use of CVD medications [9] or CVD medications used in an acute hospital setting [8, 20], whereas we were able to determine patterns of CVD medications use in the community/outpatient settings using a nationwide database that captures prescription medicines.

Patients with cancer receive multiple potentially cardiotoxic agents [21] with projections for significant increases in the future, making the management of CVD risk an important health priority. Our findings, which showed no differences in the utilisation of CVD medicines after adjusting for CVD status, suggest a relative underutilisation of potentially protective CVD medications in persons with cancer. This is concerning given that protective lifestyle interventions are also less frequently applied by cancer survivors despite their beneficial effects [22]. There remains an urgent need for the development of evidence‐based strategies to improve the management of CVD risk, including appropriate risk assessment tools, patient and provider resources, and pathways for care delivery [23].

In contrast to the lack of any difference in CVD medication use, our study demonstrated that cancer survivors with CVD accessed more medical services than the general population, emphasising the additional burden of multimorbidity and its impact on health care utilisation. While other studies have demonstrated that the presence of comorbidity is associated with a greater likelihood of specific health service use among people with cancer, previous analyses did not distinguish utilisation rates for cancer survivors living with comorbid CVD [24]. Our study provides a first step in quantifying a range of healthcare service utilisation by cancer and CVD status. Higher rates of medical services utilisation among cancer survivors with CVD may partly be a reflection of the monitoring and laboratory testing required for the management of both diseases, on top of the additional burden of symptoms or other comorbid conditions that are associated with cancer and CVD such as obesity, fatigue, and mental distress [5]. These findings may reflect genuine patient need but may also reflect inefficiencies of the health system which can potentially be addressed by better management of multimorbidity and better care integration of cardio‐oncology and survivorship care. Further studies are required to identify the specific reasons for this higher health service utilisation and to examine the impact of dedicated cardio‐oncology services on patient and health system outcomes [23].

Our study reinforces the significant impact of comorbid disease in people with and without cancer, consistent with other studies that show a positive association between comorbidity and health service use [24], highlighting the need for integrated approaches to multimorbidity management. We also found that people living outside of major cities were less likely to use medical services in both cancer and non‐cancer groups, due to barriers to accessing health services such as limited infrastructure and higher costs [24, 25].

### 4.1 Study Limitations

This study has several limitations. The information collected in the NHS, such as cancer and CVD status, was self‐reported and may be subject to response bias and measurement error. Medications dispensed as private scripts or purchased over‐the‐counter, including low‐dose aspirin (antiplatelet) are not captured in the PBS data [14], which may cause an underestimation of CVD medicine use. CVD medication might be prescribed for other conditions (aside from CVD), but we were unable to determine the specific indication. We were unable to assess the potential for varying healthcare needs based on cancer stages and time from cancer diagnosis, as this information was not collected in the survey. Additionally, the MBS data does not capture services provided to public patients in hospitals, for example, in or out‐patients, accident and emergency department visits, and non‐hospital services subsidised by private health insurance or health screening services [26].

## 5 Conclusion

In conclusion, this study comparing CVD medication use in cancer and the general population found no difference in the dispensation for any CVD medications between the two groups, but a higher rate of health service use in those with cancer and CVD. Future research should focus on better defining the causes of the increased burden of healthcare utilisation in this high‐risk population and examine whether better strategies are required to help mitigate the detrimental effects of comorbid CVD in cancer.

## Author Contributions

**Jin Quan Eugene Tan:** conceptualization (lead), formal analysis (lead), investigation (lead), methodology (lead), visualization (lead), writing – original draft (lead), writing – review and editing (lead). **Huah Shin Ng:** conceptualization (lead), formal analysis (equal), funding acquisition (lead), investigation (lead), methodology (lead), validation (lead), visualization (equal), writing – original draft (equal), writing – review and editing (equal). **Richard Woodman:** conceptualization (supporting), formal analysis (supporting), investigation (supporting), methodology (supporting), writing – review and editing (supporting). **Bogda Koczwara:** conceptualization (supporting), funding acquisition (equal), investigation (supporting), methodology (supporting), writing – original draft (supporting), writing – review and editing (supporting).

## Ethics Statement

This study was approved by Flinders University Human Ethics Low Risk Panel (#6002).

## Conflicts of Interest

The authors declare no conflicts of interest.

## Supporting information


**Table S1.** Classification of cardiovascular medications by Anatomical Therapeutic Chemical system.
**Table S2.** Description of each broad type of service categories.
**Table S3.** Frequency of cardiovascular disease medications by cancer status.
**Table S4.** The odds of dispensing of cardiovascular medications between people with and without cancer (by excluding those aged < 50 years old).
**Table S5.** Frequency and rate of medical services by cancer and cardiovascular disease (CVD) status.
**Table S6.** Medical service use by cancer and cardiovascular disease status (by excluding those aged < 50 years old).

## Data Availability

As we are not the data custodians, we are not authorised to make the data available. With the appropriate approvals, the data may be accessed through the Australian Bureau of Statistics.
