# Supplementary material for: Cardiovascular Medication and Health Service Use in Individuals With Cancer: A Retrospective Population‐Based Cohort Study
Source: Cancer Med. 2025 May 9;14(9):e70911. doi: 10.1002/cam4.70911 (PMC12062870; doi:10.1002/cam4.70911)
Supplement: Supplementary file 1 — Table S1. Classification of cardiovascular medications by Anatomical Therapeutic Chemical system. Table S2. Description of each broad type of service categories. Table S3. Frequency of cardiovascular disease medications by cancer status. Table S4. The odds of dispensing of cardiovascular medications between people with and without cancer (by excluding those aged < 50 years old). Table S5. Frequency and rate of medical services by cancer and cardiovascular disease (CVD) status. Table S6. Medical service use by cancer and cardiovascular disease status (by excluding those aged < 50 years old). [file CAM4-14-e70911-s001.docx]

**Supplementary Table 1.** Classification of cardiovascular medications by Anatomical Therapeutic Chemical system

| **Anatomical Therapeutic Chemical (ATC) system** | **Examples of medicines** |
| --- | --- |
| C01 Cardiac therapy | Cardiac glycosides e.g., digoxin  Antiarrhythmics Class I and III e.g., disopyramide, flecainide, amiodarone, sotalol  Vasodilators e.g., isosorbide dinitrate, isosorbide mononitrate, glyceryl trinitrate, nicorandil, vericiguat, perhexiline  Other cardiac preparations e.g., ivabradine |
| C02 Antihypertensives | Antiadrenergic agents centrally acting e.g., methyldopa, clonidine, moxonidine  Antiadrenergic agents peripherally acting e.g., prazosin  Agents acting on arteriolar smooth muscle e.g., hydralazine, minoxidil  Other antihypertensives for pulmonary hypertension e.g., ambrisentan bosentan, macitentan, epoprostenol, iloprost, riociguat, selexipag, sildenafil, tadalafil |
| C03 Diuretics | Low-ceiling diuretics e.g., hydrochlorothiazide, chlortalidone, indapamide  High-ceiling diuretics e.g., furosemide  Aldosterone antagonists e.g., spironolactone, eplerenone  Other potassium-sparing agents e.g., amiloride  Combination of medications that include diuretics were also included. |
| C07 Beta-blocking agents | Selective beta-blocking agents e.g., metoprolol, atenolol, bisoprolol, nebivolol  Non-selective beta-blocking agents e.g., propranolol  Alpha and beta-blocking agents e.g., labetalol, carvedilol |
| C08 Calcium channel blockers | Selective calcium channel blockers with mainly vascular effects e.g., amlodipine, felodipine, nifedipine, lercanidipine  Selective calcium channel blockers with direct cardiac effects e.g., verapamil, diltiazem  Combination of medications that include calcium channel blockers were also included. |
| C09 Agents acting on the renin-angiotensin system | Angiotensin-converting enzyme inhibitors (ACEI) e.g., captopril, enalapril, lisinopril, perindopril, ramipril, quinapril, fosinopril, trandolapril, ACEI and diuretics combinations, ACEI and calcium channel blockers combinations  Angiotensin II receptor blockers (ARBs) e.g., losartan, eprosartan, valsartan, irbesartan, candesartan, telmisartan, olmesartan, ARB and diuretics combinations, ARB and calcium channel blockers combinations, ARBs and other combinations (e.g., sacubitril with valsartan). |
| C10 Lipid modifying agents | Statins e.g., simvastatin, pravastatin, fluvastatin, atorvastatin, rosuvastatin  Fibrates e.g., gemfibrozil, fenofibrate  Bile acid sequestrants e.g., colestyramine  Other lipid modifying agents e.g., ezetimibe, alirocumab, evolocumab  Combinations of various lipid-modifying agents |
| B01 Antithrombotic agents | Vitamin K antagonists e.g., warfarin  Heparin group e.g., enoxaparin sodium, heparin  Platelet aggregation inhibitors excl heparin e.g., aspirin, clopidogrel, ticagrelor, tirofiban  Enzymes e.g., tenecteplase  Direct thrombin inhibitors e.g., dabigatran  Direct factor Xa inhibitors e.g., rivaroxaban, apixaban  Other antithrombotic agents e.g., fondaparinux |

**Supplementary Table 2**. Description of each broad type of service categories

| **BTOS categories** | **BTOS description** |
| --- | --- |
| Non-referred attendances – General Practitioner (GP)/Vocationally registered GP (VRGP) | Visits to a GP or VRGP |
| Non-referred attendances – enhanced primary care | Health assessments; GP management plans, team care arrangements and multidisciplinary care plans; GP mental health treatment plans; case conferences; domiciliary and residential medication management reviews |
| Non-referred attendances – other | Appointments at consulting rooms, or nursing homes, or hospitals where no other item applies; family group therapy, examinations by specialists in preparation for anaesthesia administration. |
| Practice nurse items | Services provided by practice nurses or Aboriginal health practitioners on behalf of a medical practitioners |
| Other allied health | Services provided by e.g., clinical psychology, physiotherapy, and other allied health professionals, under team care or shared care plan in managing those with chronic, complex needs |
| Specialist attendance | Attendances by a specialist/consultant physician |
| Obstetrics | Included obstetrics services (e.g., planning and management of pregnancy, antennal and postnatal attendances, management of labour and birth, caesarean sections and other obstetric procedures) |
| Anaesthetics | Anaesthesia for medical procedures |
| Pathology collection items | Start of patient episode through collection of a specimen |
| Pathology tests | All pathology tests (e.g., chemical, cytology, hematology, immunology, microbiology, tissue pathology, simple basic pathology tests) |
| Diagnostic imaging | Various imaging modalities (e.g., ultrasound, computed tomography, diagnostic radiography, magnetic resonance imaging, nuclear medicine imaging) for all purposes (e.g., general, obstetric and gynaecological, cardiac etc.) |
| Operations | Surgical procedures for various specialities such as general, vascular, plastics and reconstructive etc. |
| Assistance at operations | 11 item codes for which assistance was needed during an operation |
| Optometry | Initial and follow-up consultations, appointments for contact lenses, etc. |
| Radiotherapy and therapeutic nuclear medicine | Various radiation oncology services (e.g., megavoltage, brachytherapy etc.) and therapeutic nuclear medicine (e.g., administering radioisotopes or iodine etc.) |
| Other Medicare Benefits Schedule (MBS) services | Other services not listed elsewhere |

References:

Callander E, Bates N, Lindsay D, Larkins S, Topp SM, Cunningham J, et al. Long-term out of pocket expenditure of people with cancer: comparing health service cost and use for indigenous and non-indigenous people with cancer in Australia. Int. J. Equity Health. 2019;18(1):32.

Australian Institute of Health and Welfare (AIHW). Medicare Benefits Scheme funded services over time. 2023. Accessed at https://www.aihw.gov.au/reports/health-welfare-expenditure/mbs-funded-services-over-time/contents/technical-notes/measures-used-in-the-report on January 24, 2024.

**Supplementary Table 3**. Frequency of cardiovascular disease medications by cancer status

| **Types of cardiovascular medications** | **Cancer**  **N=1828 (%)** | **Non-cancer**  **N= 7505 (%)** |
| --- | --- | --- |
| Any cardiovascular medications | 1101 (60) | 2361 (31) |
| ATC C01 (cardiac therapy) | 106 (6) | 153 (2) |
| ATC C02 (antiadrenergic) | 63 (3) | 125 (2) |
| ATC C03 (diuretics) | 269 (15) | 471 (6) |
| ATC C07 (beta-blocking agents) | 239 (13) | 490 (7) |
| ATC C08 (calcium channel blockers) | 352 (19) | 654 (9) |
| ATC C09 (agents acting on the renin-angiotensin system) | 734 (40) | 1525 (20) |
| ATC C10 (lipid modifying agents) | 675 (37) | 1327 (18) |
| ATC B01 (anti-thrombotic agents) | 248 (14) | 376 (5) |

Key: ATC, Anatomical Therapeutical Chemical

**Supplementary Table 4.** The odds of dispensing of cardiovascular medications between people with and without cancer (by excluding those aged <50 years old)

| **Types of cardiovascular medications** | **Adjusted odds ratio (95% CI)**  **Reference group: non-cancer** |
| --- | --- |
| Any cardiovascular medications  Model A  Model B | 1.24 (1.08-1.43)  1.10 (0.94-1.28) |
| ATC C01 (cardiac therapy)  Model A  Model B | 1.13 (0.85-1.48)  1.02 (0.77-1.36) |
| ATC C02 (antiadrenergic)  Model A  Model B | 0.99 (0.70-1.39)  0.94 (0.66-1.32) |
| ATC C03 (diuretics)  Model A  Model B | 1.20 (1.00-1.44)  1.12 (0.93-1.35) |
| ATC C07 (beta-blocking agents)  Model A  Model B | 1.05 (0.87-1.27)  0.96 (0.79-1.17) |
| ATC C08 (calcium channel blockers)  Model A  Model B | 1.11 (0.95-1.31)  1.04 (0.88-1.23) |
| ATC C09 (agents acting on the renin-angiotensin system)  Model A  Model B | 1.08 (0.94-1.23)  0.95 (0.82-1.10) |
| ATC C10 (lipid modifying agents)  Model A  Model B | 1.08 (0.95-1.24)  1.01 (0.88-1.17) |
| ATC B01 (anti-thrombotic agents)  Model A  Model B | 1.29 (1.06-1.56)  1.20 (0.99-1.47) |

Key: ATC, Anatomical Therapeutic Chemical; CI, confidence interval.

Model A: The logistic regression model was adjusted for sociodemographic characteristics (sex, age group, marital status, country of birth, geographical location, education level, employment status, socioeconomic status), and lifestyle factors (body mass index, smoking status, alcohol intake, vegetable/fruit intake, and physical activity), as well as number of other current health conditions.

Model B: The model was adjusted using the same adjustment variables as model A with the addition of self-reported cardiovascular disease status.

**Supplementary Table 5**. Frequency and rate of medical services by cancer and cardiovascular disease (CVD) status.

|  | **Disease status/ total person-years (py) of follow-up** | | | |
| --- | --- | --- | --- | --- |
| **Types of services** | **Cancer and**  **CVD/**  **1141 py** | **Non-cancer and CVD/**  **2477 py** | **Cancer and**  **no CVD/**  **687 py** | **Non-cancer and no CVD/**  **5028 py** |
| **Any medical services**  Sum of counts  Crude rate (100py) | 47890  4197.20 | 76257  3078.60 | 16483  2399.27 | 77604  1543.44 |
| **Pathology tests**  Sum of counts  Crude rate (100py) | 13184  1155.48 | 20332  820.83 | 4271  621.69 | 21484  427.29 |
| **Non-referred GP/VRGP**  Sum of count  Crude rate (100py) | 12522  1097.46 | 22991  928.18 | 4548  662.01 | 24728  491.81 |
| **Pathology collection items**  Sum of count  Crude rate (100py) | 5531  484.75 | 8777  354.34 | 1992  289.96 | 10729  213.39 |
| **Specialist attendance**  Sum of count  Crude rate (100py) | 4660  408.41 | 6174  249.25 | 1439  209.46 | 4229  84.11 |
| **Diagnostic imaging**  Sum of count  Crude rate (100py) | 2770  242.77 | 4708  190.07 | 1057  153.86 | 4404  87.59 |
| **Operations**  Sum of count  Crude rate (100py) | 1818  159.33 | 1765  71.26 | 692  100.73 | 1521  30.25 |
| **Other MBS**  Sum of count  Crude rate (100py) | 1734  151.97 | 2425  97.90 | 426  62.01 | 1333  26.51 |
| **Other allied health**  Sum of count  Crude rate (100py) | 1440  126.21 | 2567  103.63 | 460  66.96 | 2603  51.77 |
| **Non-referred- enhanced primary care**  Sum of count  Crude rate (100py) | 1229  107.71 | 2202  88.90 | 319  46.43 | 1805  35.90 |
| **Optometry**  Sum of count  Crude rate (100py) | 926  81.16 | 1679  67.78 | 427  62.15 | 2101  41.79 |
| **Practice nurse items**  Sum of count  Crude rate (100py) | 522  45.75 | 784  31.65 | 73  10.63 | 292  5.81 |
| **Anaesthetics**  Sum of count  Crude rate (100py) | 537  47.06 | 886  35.77 | 185  26.93 | 614  12.21 |
| **Non-referred- other**  Sum of count  Crude rate (100py) | 308  26.99 | 621  25.07 | 96  13.97 | 857  17.04 |

Key: CVD, cardiovascular disease; py, person-years

**Supplementary Table 6**. Medical service use by cancer and cardiovascular disease status (by excluding those aged <50 years old)

| **Medical service categories by cancer and comorbidity status** | **Adjusted rate ratio^a^**  **(95% confidence interval)** |
| --- | --- |
| **Any medical services**  Non-cancer, No CVD  Cancer & CVD  Non cancer & CVD  Cancer & No CVD | Reference  2.03 (1.90-2.18)*  1.66 (1.57-1.76)*  1.42 (1.31-1.54)* |
| **By types of services:** | |
| **Other Medicare Benefits Schedule items**  Non-cancer, No CVD  Cancer & CVD  Non-cancer & CVD  Cancer & No CVD | Reference  3.05 (2.57-3.63)*  2.42 (2.08-2.82)*  1.62 (1.30-2.01)* |
| **Specialist attendance**  Non-cancer, No CVD  Cancer & CVD  Non-cancer & CVD  Cancer & No CVD | Reference  2.86 (2.48-3.29)*  2.07 (1.83-2.34)*  1.85 (1.56-2.19)* |
| **Operations**  Non-cancer, No CVD  Cancer & CVD  Non-cancer & CVD  Cancer & No CVD | Reference  2.41 (2.05-2.84)*  1.41 (1.22-1.63)*  2.13 (1.76-2.58)* |
| **Practice nurse items**  Non-cancer, No CVD  Cancer & CVD  Non-cancer & CVD  Cancer & No CVD | Reference  2.35 (1.83-3.03)*  2.21 (1.76-2.77)*  1.14 (0.80-1.61) |
| **Pathology tests**  Non-cancer, No CVD  Cancer & CVD  Non-cancer & CVD  Cancer & No CVD | Reference  2.32 (2.11-2.54)*  1.79 (1.65-1.93)*  1.41 (1.26-1.58)* |
| **Pathology collection items**  Non-cancer, No CVD  Cancer & CVD  Non-cancer & CVD  Cancer & No CVD | Reference  2.09 (1.92-2.28)*  1.60 (1.49-1.73)*  1.37 (1.23-1.52)* |
| **Diagnostic imaging**  Non-cancer, No CVD  Cancer & CVD  Non-cancer & CVD  Cancer & No CVD | Reference  1.98 (1.74-2.24)*  1.75 (1.57-1.95)*  1.48 (1.27-1.72)* |
| **Anaesthetics**  Non-cancer, No CVD  Cancer & CVD  Non-cancer & CVD  Cancer & No CVD | Reference  1.74 (1.36-2.22)*  1.70 (1.37-2.10)*  1.35 (1.01-1.82)* |
| **Other allied health**  Non-cancer, No CVD  Cancer & CVD  Non-cancer & CVD  Cancer & No CVD | Reference  1.74 (1.35-2.24)*  1.60 (1.30-1.97)*  1.25 (0.93-1.70) |
| **Non-referred – enhanced primary care** Non-cancer, No CVD  Cancer & CVD  Non-cancer & CVD  Cancer & No CVD | Reference  1.75 (1.50-2.04)*  1.71 (1.50-1.95)*  1.16 (0.95-1.41) |
| **Non-referred- GP/VRGP**  Non-cancer, No CVD  Cancer & CVD  Non-cancer & CVD  Cancer & No CVD | Reference  1.60 (1.50-1.69)*  1.49 (1.42-1.57)*  1.19 (1.11-1.28)* |
| **Non-referred- other**  Non-cancer, No CVD  Cancer & CVD  Non-cancer & CVD  Cancer & No CVD | Reference  1.43 (0.96-2.15)  1.39 (0.99-1.94)  0.93 (0.58-1.52) |
| **Optometry**  Non-cancer, No CVD  Cancer & CVD  Non-cancer & CVD  Cancer & No CVD | Reference  1.18 (1.07-1.32)*  1.14 (1.04-1.24)*  1.09 (0.96-1.24) |

Key: CVD, cardiovascular disease; GP, general practitioner; VRGP; vocationally registered general practitioner.

CVD status was determined by self-reported as having a current CVD and/or with a dispensing record of CVD medications within one year from the date of survey completion. Number of people in each group as follows: Non-cancer and no CVD (n = 1674/5258; 32%), cancer and CVD (n = 1091/5258; 21%), non-cancer and CVD (n=1975/5258; 38%) and cancer and no CVD (n=518/5258; 10%).

^a^ The negative binomial regression was adjusted for sociodemographic characteristics (sex, age, marital status, country of birth, geographical location, education level, employment status, socioeconomic status), and lifestyle factors (body mass index, smoking status, alcohol intake, vegetable/fruit intake, and physical activity), as well as number of other current health conditions (excluding cancer).

*p-value <0.05
